# Advent of three-dimensional sediment exploration reveals Ediacaran-Cambrian ecosystem transition

**DOI:** 10.1126/sciadv.adx9449

**Published:** 2025-10-29

**Authors:** Zhe Chen, Yarong Liu

**Affiliations:** ^1^ State Key Laboratory of Palaeobiology and Stratigraphy, Nanjing Institute of Geology and Palaeontology, Chinese Academy of Sciences, Nanjing 210008, China.; ^2^ University of Chinese Academy of Sciences, Beijing 100049, China.

## Abstract

Trace fossils offer critical insights into animal diversification and ecosystem evolution during the Neoproterozoic-Phanerozoic transition. *Treptichnus* represents the earliest known three-dimensional burrow system, which co-occurred with Ediacara-type fossils in the Shibantan assemblage (~550 to 543 million years ago). Integration with *Lamonte* and tadpole-like traces reveals important behavioral innovations from simple horizontal locomotion to complex sediment penetration, thereby bridging ecological and behavioral transitions across the Ediacaran-Cambrian boundary. The advent of three-dimensional sediment exploration fundamentally altered benthic ecodynamics and disrupted microbial mat stability. This shift reflects a fundamental restructuring of infaunal habitats, which temporally coincided with the decline of matground-related Ediacaran macro-organisms. These innovations laid the foundation for Phanerozoic animal-sediment interactions, catalyzing a pivotal ecological transition predating yet enabling the Cambrian Explosion.

## Teaser

Three-dimensional burrowing triggered Ediacaran ecosystem shift, catalyzing animal diversification before the Cambrian Explosion.

## INTRODUCTION

Trace fossils provide critical insights into metazoan diversification and ecosystem shift during the Neoproterozoic-Paleozoic transition, approximately 539 million years (Ma) ago (*1*, *2*). Ediacaran trace fossils are predominantly characterized by simple horizontal trails and shallow burrows near the sediment-water interface, reflecting limited behavioral complexity (*3*–*5*). In contrast, Cambrian strata record a dramatic proliferation of ichnofossil diversity, including complex branching patterns and vertical sediment penetration, signaling the emergence of sophisticated animal behaviors (*2*, *6*, *7*). Notably, pervasive vertical bioturbation, a hallmark of Phanerozoic ecosystems, first appears near the Ediacaran-Cambrian boundary (*5*). Among these innovations, *Treptichnus* occupies a pivotal position in evolutionary ichnology. This ichnogenus, characterized by three-dimensional burrow systems, represents the earliest evidence of complex subsurface exploration (*8*–*10*). Although the first appearance of *Treptichnus pedum* traditionally marks the Ediacaran-Cambrian boundary (*11*), the discovery of this trace fossil several meters below the Global Stratotype Section and Point in Newfoundland (*12*) complicates its stratigraphic significance. Researchers have further identified *Treptichnus* or treptichnid-like traces in uppermost Ediacaran strata (*1*, *4*, *13*, *14*). Tarhan *et al.* (*15*) reported the oldest putative treptichnids from the late Ediacaran Dunfee Member (Deep Spring Formation, Nevada), which may predate the end of Ediacaran by 1 to 10 million years (Myr), although more precise geochronological constraints are still lacking. The earliest definitively dated treptichnids appear in the Nasep-Huns transition (Schwarzrand Subgroup, Namibia), with recent radiometric ages constraining these horizons to 540.83 Ma (*16*, *17*).

The Shibantan assemblage (~550 to 543 Ma) in the Yangtze Gorges area of South China offers unique insights into this critical transition. Within the Dengying Formation, this lagerstätte preserves an exceptional co-occurrence of Ediacara-type soft-bodied fossils (e.g., fronds and tubular organisms) with diverse trace fossils (*18*–*23*), including three-dimensional burrow systems such as *Treptichnus*, *Lamonte*, and tadpole-like burrows. These ichnofossils document the advent of vertical sediment exploration, a behavioral breakthrough marked by systematic probing of microbial-mat substrates. The Shibantan traces provide independent evidence for early bilaterian evolution, revealing an increase in ecological tiering and behavioral complexity before the Cambrian explosion. The Shibantan assemblage encapsulates a key transitional phase, in which three-dimensional sediment exploration reshaped benthic ecologies, bridging the ecological simplicity of the Ediacaran with the dynamic ecosystems of the Cambrian.

## RESULTS

### Stratigraphy, age constraints, and fossils

**Fig. 1.**
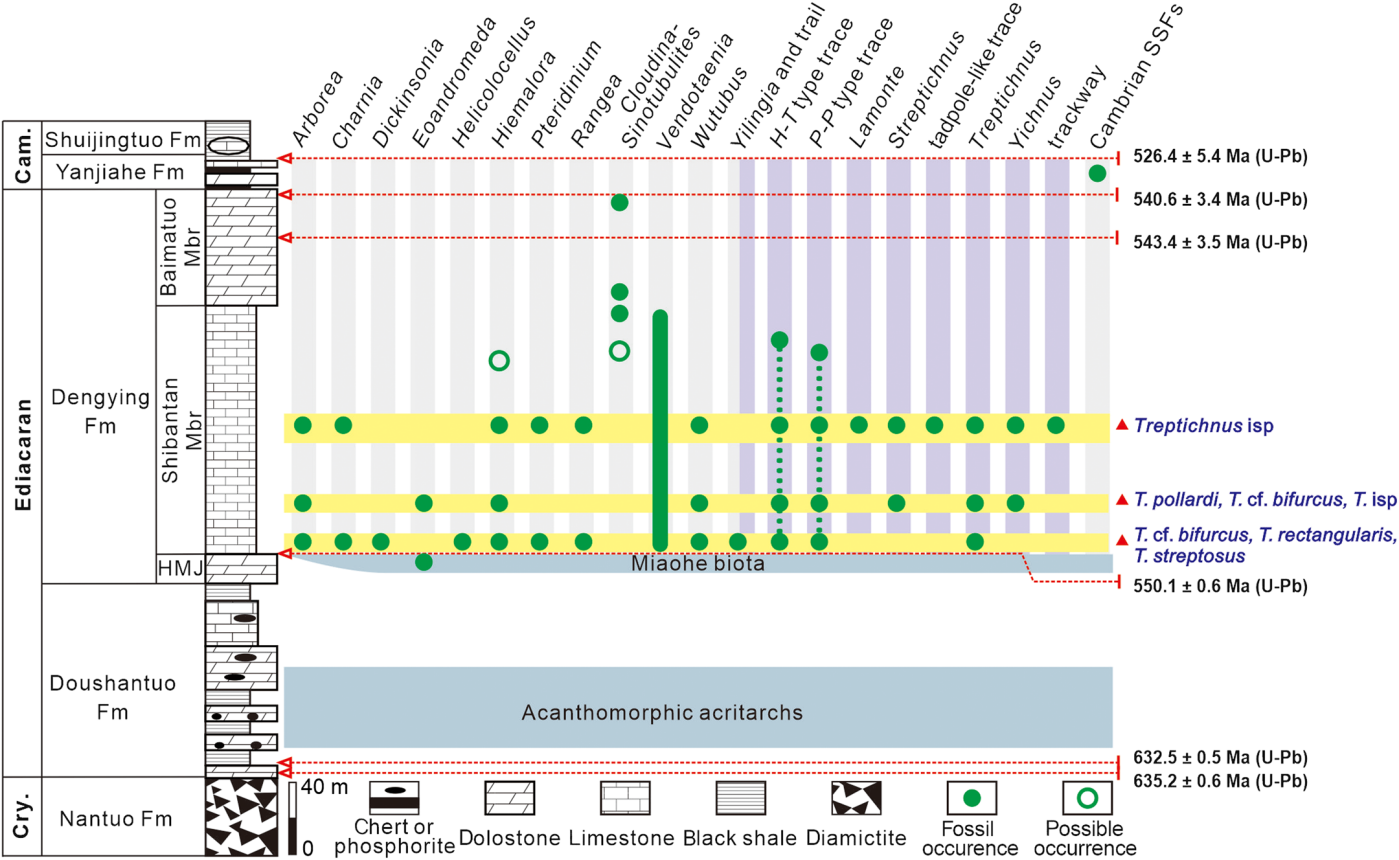
The stratigraphic column and stratigraphic distribution of key fossils in the Shibantan assemblage. Three excavated fossiliferous horizons are denoted by yellow bands, and trace fossils are denoted by purple columns. Cry, Cryogenian; Cam, Cambrian; Fm, Formation; Mbr, Member; HMJ, Hamajing Member; SSFs, small shelly fossils; *H*-*T* type trace, *Helminthoidichnites*-*Torrowangea* type trace; *P*-*P* type trace, *Planolites*-*Palaeophycus* type trace. Zircon U-Pb ages from published data (*28*, *29*, *35*, *79*).

The Ediacaran System is well exposed in the Yangtze Gorges area, consisting of the Doushantuo (inner shelf lagoon deposits) and Dengying formations (shallow carbonate platform deposits) (*24*). The Ediacaran System overlies the Cryogenian Nantuo Formation diamictite and underlies the lower Cambrian Yanjiahe Formation that contains small shelly fossils and the *Asteridium*-*Comasphaeridium*-*Heliosphaeridium* microfossil assemblage (*25*, *26*). The upper Ediacaran Dengying Formation is subdivided into three members in ascending order: the Hamajing, Shibantan, and Baimatuo members (Fig. 1). The Shibantan Member consists of dark gray, thin-bedded, laminated micritic limestone deposited in subtidal environments (*21*).

The Shibantan Member (~550 to 543 Ma) constitutes a critical taphonomic window, containing soft-bodied Ediacara-type fossils (e.g., *Charnia*, *Hiemalora*, *Pteridinium*, *Dickinsonia*, *Arborea*, *Rangea*, and *Eoandromeda*), algae, and trace fossils (*18*–*23*). Taxonomically, the Shibantan assemblage seems to be intermediate between the White Sea assemblage (~558 to 550 Ma) and the Nama assemblage (~550 to 539 Ma) (*18*, *27*). Geochronologically, the Shibantan assemblage is constrained between ~550 Ma and 543 Ma (*28*, *29*). An ash bed from the top of the Miaohe Member yields a zircon U-Pb age of 550.1 ± 0.6 Ma (*28*). The Miaohe Member was traditionally correlated with the Member IV of the Doushantuo Formation (*30*). However, recent studies on the regional litho- and sequence stratigraphy suggest that the Miaohe Member is likely to be equivalent to the lower Shibantan Member (*31*, *32*). Additional age constraints derive from the biostratigraphic ranges of *Cloudina* and *Sinotubulites*. The occurrence of *Cloudina* is between >547.36 and ~538.58 Ma in the Nama Group (*16*, *17*, *33*, *34*). In the Yangtze Gorges, the reported occurrences of *Cloudina* or *Sinotubulites* span four stratigraphic horizons, including the uppermost Baimatuo Member (*35*), the lower Baimatuo Member (*36*), the transition sequences between the Baimatuo Member and Shibantan Member (*37*), and the upper Shibantan Member (*38*, *39*). These data confirm that the Shibantan Member began depositing approximately 550 Ma. Zircon U-Pb ages of 543.4 ± 3.5 Ma from the middle (*29*) and 540.6 ± 3.4 Ma from the top of the overlying Baimatuo Member provide the minimum age constraints of the Shibantan Member (*35*).

The Shibantan assemblage represents a distinctive late Ediacaran ecosystem, yielding an ichnofauna from simple surficial traces (such as *Helminthoidichnites*-*Torrowangea* type and *Planolites*-*Palaeophycus* type) to complex three-dimensional burrow systems (such as *Treptichnus*, *Lamonte*, and tadpole-like traces). We collected the trace fossils documented herein from three horizons (Fig. 1) near Wuhe Village, Yangtze Gorges area (30° 47′ 10″ to 30° 47′ 27″ N, 111° 02′ 47″ to 111° 03′ 23″ E). (i) Basal horizon (0 to 5 m above the base): dominated by Ediacara-type body fossils (Charnids, *Arborea*, *Yilingia*, *Hiemalora*, *Helicolocellus*, *Wutubus*, and *Aspidella*-type discs). Trace fossils are sparse, primarily consisting of *Helminthoidichnites*. Co-occurrence of body and trace fossils can occur on shared bedding planes. (ii) Lower horizon (18 to 25 m): overwhelmingly covered by *Yangtziramulus*-like structures (*18*). Both body fossils (e.g., *Arborea*, *Wutubus* and *Aspidella*-type disc) and trace fossils (e.g., *Helminthoidichnites*) exhibit the lowest abundance in these three horizons. (iii) Middle horizon (~70 m): proliferation of complex burrows, for example, *Lamonte* covering 20 to 40% bedding planes locally (*21*); with *Wutubus*, *Pteridinium*, *Rangea*, *Hiemalora*, and *Aspidella*-type discs. *Helminthoidichnites*-*Torrowangea* and *Planolites*-*Palaeophycus* dominate trace fossils throughout the member. *Treptichnus* is rare (11 specimens total), sparsely distributed on bedding surfaces. In contrast, *Lamonte* and tadpole-like traces are abundant in the middle horizon. Ediacara-type fossils rarely occur on trace-dense surfaces except for *Aspidella*-type discs and *Wutubus*, which occasionally persist in moderately disturbed matgrounds.

### *Treptichnus* burrow system

**Fig. 2.**
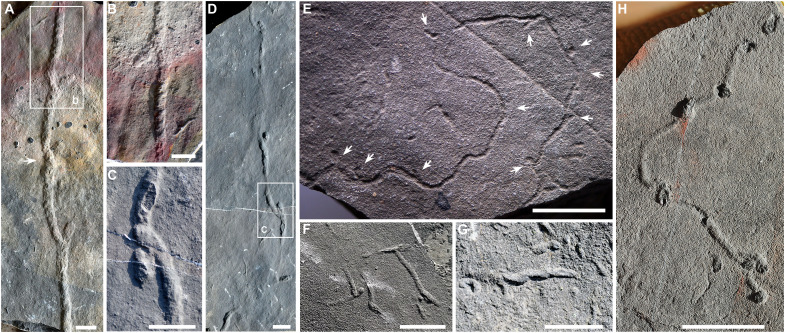
*Treptichnus* in the Shibantan assemblage in the Wuhe area. (**A** and **B**) *Treptichnus streptosus* isp. nov., the segments overlap at the joints, the arrow indicating the most noticeable overlap, NIGP-205651, from the basal Shibantan Member; (B) enlargement of the rectangle in (A), noting the regular inclined ridges. (**C** to **E**) *T*. cf. *bifurcus*; (C) enlargement of the rectangle in (D), showing the overlap of segments, NIGP-205652, from the basal Shibantan Member; (E) segment with short projections (arrows), NIGP-205653, from the lower Shibantan Member. (**F**) *T. rectangularis*, segments connected in nearly right angles, NIGP-205654, from the basal Shibantan Member. (**G**) *T*. isp., imbricate arranged burrow segments, field photo from the middle Shibantan Member. (**H**) *T. pollardi*, smooth burrow segments with bulbous joint, NIGP-205655. Scale bars represent 2 cm.

We identified five *Treptichnus* ichnospecies within the Shibantan assemblage and differentiated them through branching architecture and segment morphology (Fig. 2; detailed descriptions in the Supplementary Materials). *Treptichnus* is a well-studied trace fossil genus, characterized by a series of connected burrow segments forming zigzag or meandering patterns, often with short projections at segment junctions (*9*). However, species-level distinctions within this genus remain challenging due to morphological variability (*9*, *40*). Although three-dimensional structures are not visible in the bed plane, the specimens from Wuhe exhibit distinct meanders and horizontally arranged zigzag segments, consistent with the morphology of *Treptichnus*. We assign these traces to *T*. cf. *bifurcus*, *T. pollardi*, *T. rectangularis*, *T*. isp., and erected a new ichnospecies *T. streptosus* isp. nov.

*Treptichnus* cf. *bifurcus* (Fig. 2, C to E) displays straight, subhorizontal burrows that zigzag or meander, featuring short projections between elongated burrow segments. These specimens (Fig. 2, C to E) exhibit *T*. *bifurcus*–like short twig projections at zigzagging burrow junctions but display irregular alternations, unlike the diagnostic rhythmic pattern of *T*. *bifurcus* (*9*, *41*). Tentatively, we assign them to *T*. cf. *bifurcus*. *T. pollardi* (Fig. 2H and fig. S1) displays horizontal cylindrical burrow segments connected in an alternating pattern, with bulbous structures at the zigzag corners and no lateral projections (*9*). The burrow segments of *T. rectangularis* (Fig. 2F) exhibit expanding ends, with variable angles between connected burrow segments that tend to be right-angled (*42*). A burrow (Fig. 2G), composed of an imbricated arrangement of short segments lacking obvious protrusions, we tentatively assigned it to *T*. isp. The newly proposed ichnospecies, *T. streptosus* isp. nov. (Fig. 2A), is a subhorizontal burrow consisting of successively superimposed burrow segments with short projections. Its diagnostic feature is the presence of distinctive inclined ridges on the surface of the burrow segments. *T. streptosus* represents a relatively large burrow system, with burrow diameters exceeding 1 cm. The burrow elements of this new ichnospecies share similarities with *Streptichnus narbonnei*, which occurs just above the Ediacaran-Cambrian boundary in Namibia, dated between 538.99 ± 0.21 and 538.58 ± 0.19 Ma (*34*, *43*).

*Treptichnus* represents a major innovation in burrowing behavior, characterized by a distinctive architecture resulting from successive probing activities (*44*, *45*). The zigzag patterns reflect periodic changes in direction, demonstrating an efficient and sophisticated strategy for deposit feeding (*9*, *46*). Burrow segments of *Treptichnus* often terminate in bulbous or projection structures, which most likely feature openings to communicate with the sediment surface. Fossil and neoichnological evidence suggest that various organisms, including worm-form priapulid (*44**,* *45*), insect larvae (*40*, *46*–*48*), and insects (*49*), could produce *Treptichnus* and treptichnid-like structures. Given its long stratigraphic range and broad environmental distribution, multiple trace makers likely produced *Treptichnus* (*8*, *40*). Despite uncertain taxonomic affinities, the *Treptichnus* (*T. pedum*) producer was a bilaterian animal having sensory neural networks (*50*).

### Compound traces with vertical structure

**Fig. 3.**
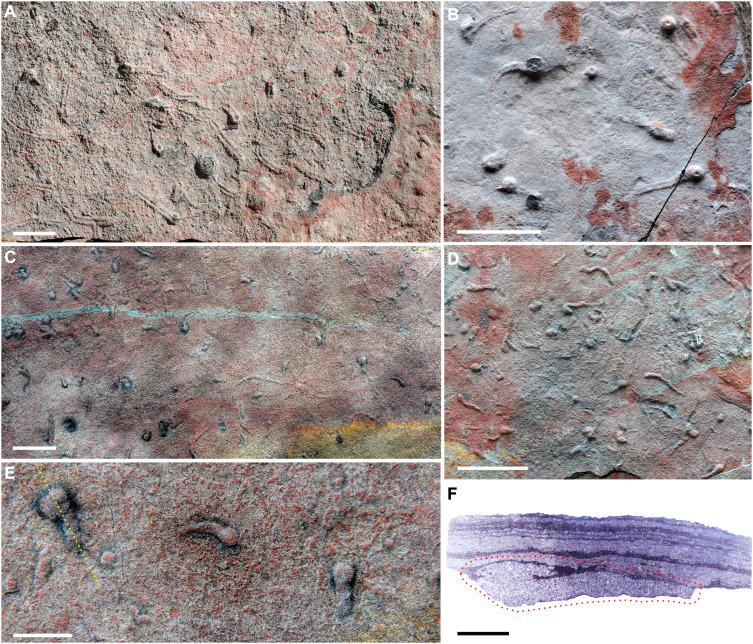
Trace fossils with vertical structure in the Shibantan assemblage. (**A** and **B**) *Lamonte*, noting depression in the center of vertical plug-shaped structures. (**C** to **F**) Tadpole-like trace; (E) close up of tadpole-like trace; (F) longitudinal section of the left specimen along the yellow dot line in (E), trace within red dot line. All the specimens are on the bottom surface in situ and were collected from the middle Shibantan Member. Scale bars represent 5 cm for (A) to (D), 2 cm for (E), and 5 mm for (F).

The Shibantan assemblage preserves several trace fossils exhibiting horizontal or subhorizontal burrows integrated with vertical components. As illustrated in Fig. 3, these traces are preserved as full relief and have been described as *Lamonte trevallis* (*21*) (Fig. 3, A and B) and tadpole-like traces (Fig. 3, C to F) (*20*). *Lamonte* is characterized by the interconnection of the bilobate horizontal tunnel and vertical burrow, occasionally accompanied by surface tracks. The co-occurrence of these three interconnected trace types suggests distinct behaviors of the trace makers, representing undermat feeding or locomotion, temporary dwelling, and surface locomotion, respectively (*19*, *21*). The tadpole-like trace comprises a narrow, subhorizontal burrow connected to an expanding bulb structure at one end. The bulbs are noticeably larger than the associated burrows, and the burrow itself is subhorizontal, unlined and tapers gradually toward the opposite end. In cross-section, the bulbs clearly truncated microbial mat (Fig. 3F), indicating openings at the sediment surface. Morphologically, the tadpole-like trace resembles a J-shaped burrow or a segment of treptichnids, distributed randomly within the sediment. This morphology suggests that the trace-making animal penetrated the sediment to exploit organic particles, and has been interpreted as a feeding structure (*20*). The bulb represents vertical probing, while the burrow reflects horizontal locomotion or foraging activities.

The interconnected structures of *Lamonte* and tadpole-like traces represented compound traces, resulting from the changing behavior of a single producer (*51*). Although some studies propose cnidarian-grade animals exhibited both vertical and horizontal movements on the Ediacaran seafloor (*52*), *Lamonte* and tadpole-like traces are unlikely to be produced by cnidarians. Instead, their trace makers created undermat burrows rather than surface trails typical of cnidarians (*53*, *54*). These animals exhibited both vertical and subhorizontal burrowing capabilities. Notably, *Lamonte*’s distinct bilobate structure strongly suggests a bilaterian producer, underscoring the emergence of complex behaviors in late Ediacaran ecosystems (*19*, *21*).

## DISSCUSSION

### The advent of three-dimensional sediment exploration

**Fig. 4.**
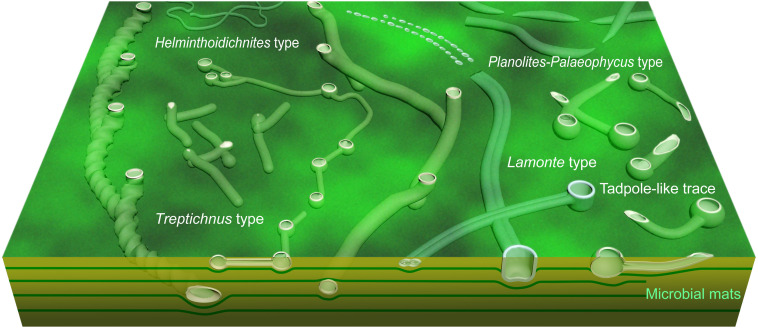
Schematic illustration of trace fossils in the Shibantan assemblage. Artwork by Z. Chen

The Ediacaran Period (~635 to 539 Ma) witnessed the emergence of metazoan-sediment interaction, with definitive evidence of bioturbation appearing after ~560 Ma (*55*–*57*). Early innovations were limited to horizontal surface trails and equilibrium structures produced by cnidarian-grade organisms (*52*–*54*). A global increase in trace fossil abundance and complexity after ~555 Ma [see summarized in (*56*, *57*)] set the stage for more advanced behaviors. By ~550 to 543 Ma, the Shibantan assemblage in South China preserved one of the earliest systematic three-dimensional sediment explorations through vertically tiered burrow systems, signaling a pivotal shift in animal behavior (Fig. 4).

The Shibantan assemblage documents the development of complex behaviors prior to the Cambrian. While dominated by simple horizontal or subhorizontal burrows (e.g., *Helminthoidichnites* and *Planolites*), it preserved three groups of trace fossils with vertical structures, including *Treptichnus*, *Lamonte*, and tadpole-like traces. Although isolated vertical structures (such as *Bergaueria*) occurred in the older strata of Precambrian [see discussions in (*3*, *56*, *58*)], they typically represent solitary behaviors, such as resting or passive responses to sediment input (*52*). In contrast, the combination of vertical and horizontal structures in the Shibantan traces suggests more advanced behavioral strategies related to food gathering.

These trace fossils from Wuhe demonstrate that Ediacaran animals began exploring sediments in three dimensions, moving beyond simple undermat horizontal burrowing. Despite some debates suggesting that *Lamonte* was confined to microbial mats without strict penetration of the underlying sediments (*56*), its vertical burrows cut through multiple microbial mats and extended deeper than the associated horizontal burrows. In particular, *T. streptosus* isp. nov. (Fig. 2, A and B) and *T.* cf. *bifurcus* (Fig. 2, C and D) display vertically stacked burrow segments, indicating penetration depth of the trace at least twice the burrow diameter, exceeding 2 cm. Regardless of penetration depth, the presence of traces with vertical structures represents a fundamental shift in infaunal habitats, and also likely had a profound impact on the marine ecosystem (*59*, *60*).

### Ecological and evolutionary implications

The integration of vertical structures within the Shibantan assemblage represents a transformative advancement in bioturbation behavior during the late Ediacaran. Unlike simple horizontal burrows restricted to the sediment-water interface, vertical burrows penetrate deeper into the substrate, reflecting a more complex and dynamic interaction between organisms and their environment (*61*). This shift from horizontal to vertical bioturbation marks a critical evolutionary innovation in animal behavior and ecosystem engineering (*62*).

The zigzag or meandering patterns of *Treptichnus* indicate active probing and systematic exploration of the sediment. These behaviors enabled trace makers to exploit organic matter at greater depths, shifting from simple near-surface grazing to structured subsurface penetration. The bioturbation associated with *Treptichnus*, *Lamonte* and tadpole-like traces had profound ecological implications. These complex bioturbation behaviors profoundly altered sediment properties by creating vertical and subhorizontal burrows that enhanced physical mixing and biogeochemical cycling (*60*, *63*). Despite intensified ecosystem engineering in the Shibantan assemblage, pervasive sediment oxygenation remained implausible under contemporaneous low oxygen conditions. Modelling confirms that redox impacts of bioturbation are environmentally contingent, with early biomixers unable to oxygenate shallow-to-deep sediment tiers in Ediacaran (*64*). However, these bioturbators likely influenced other critical biogeochemical cycles, such as phosphorus, organic carbon, and sulfur dynamics (*65*–*67*), while enhanced nutrient mixing facilitated complex benthic ecosystem development (*60*, *68*). This penetrative bioturbation near the Proterozoic-Phanerozoic transition represents a first-order ecological revolution (*4*, *69*). *Treptichnus* fossils underscore pivotal innovations in bilaterian behavioral strategies that led to substrate transformation, despite terminal Ediacaran oxygenation constraints.

The emergence of complex bioturbation behaviors, including the first treptichnid-like burrows, in the Shibantan assemblage (~550 to 543 Ma) temporally coincides with the first major extinction pulse of Ediacara-type organisms during the White Sea-Nama transition (~550 Ma) (*70*–*72*). This is powerfully illustrated by the co-occurrence of these innovative trace makers with the last appearance of *Dickinsonia* (*23*), an archetypal White Sea assemblage genus that barely crosses into the Nama assemblage and is emblematic of this first extinction pulse. This stratigraphic alignment suggests a potential link between the rise of infaunal bioturbators and biotic turnover, possibly involving competition, predation, and metazoan diversification (*14*, *71*–*73*). A pronounced negative correlation exists between trace fossil density and Ediacara-type fossil abundance: minimally bioturbated basal horizons (0 to 5 m) preserve abundant body fossils, whereas intensely bioturbated middle horizons (~70 m) exhibit trace fossil dominance [with *Lamonte* covering 20 to 40% of surfaces (*21*)] and a decrease in Ediacaran forms. While typical Ediacaran fossils, including fronds, discs, and tubular forms, persist on nonbioturbated surfaces within these middle horizons, suggesting overlapping environmental preferences, their scarcity on heavily bioturbated surfaces implies local exclusion. We therefore interpret the observed taphonomic segregation as a result of local biotic interactions, including matground disruption by trace producers and competitive exclusion in shared habitats.

This interpretation is consistent with the biotic replacement model (*15*, *71*, *72*), which attributes the White Sea–Nama transition to the impact of ecosystem engineers like bioturbators. However, the extent to which biotic processes alone drove this extinction remains uncertain, as direct evidence for antagonistic interactions is minimal (*71*). Alternatively, the catastrophic extinction model posits that a major environmental perturbation (e.g., changes in oxygen) led to the rapid loss of Ediacara taxa (*70*). The Shibantan evidence contributes to this debate by demonstrating that local ecosystem engineering and biotic interactions likely played a role, even if a sole causative mechanism remains elusive.

Subsequently, the second extinction pulse at the Ediacaran-Cambrian boundary (~539 Ma) culminated in the collapse of residual Ediacara biota, triggered by synergistic environmental perturbations, including extensive rift-related volcanism and systemic ecological overhaul (*63*, *71*, *74*). *Treptichnus*-associated bioturbation, although locally rare in Shibantan, catalyzed Cambrian diversification by destabilizing microbial mats and creating heterogeneous microhabitats for niche partitioning (*73*, *75*, *76*). Concurrently, predation pressure and spatial competition from mobile taxa likely drove the extinction of sessile Ediacaran forms adapted to stable matground ecologies (*76*–*78*).

Thus, the Shibantan assemblage captures a pivotal moment in Earth’s history, documenting the transition from a simple horizontal excursion to active three-dimensional sediment exploration, and from the Ediacaran matground-dominated ecosystem to Cambrian mixground substrate. This shift, driven by bilaterian innovation, bridged the ecological divide between limited Ediacaran bioturbation and the dynamic Phanerozoic biosphere.

### Systematic paleontology

#### Treptichnus

##### Diagnosis

Simple or zigzag, straight, or curved segments associated with vertical or oblique tubes comprising a three-dimensional burrow system. Joined points of segments exhibit small pits or short twig-like projections [from (*9*)].

*Treptichnus streptosus* isp. nov. (Fig. 2A)

##### Diagnosis

Subhorizontal burrow is composed of successively superimposed burrow segments, with short projections. The burrow surface shows distinctive, regular inclined ridges.

##### Holytype

NIGP-205651, Fig. 2A.

##### Etymology

From the Greek *streptos*, meaning twisted.

##### Description

The specimen morphology shows a certain degree of regularity. The subhorizontal burrow is composed of four successively superimposed burrow segments. The burrow is slightly curved, about 10 mm in width and 36 cm in length. The burrow segments are slightly curved, with nearly constant length, about 11 cm. The projections protrude from the concave side of the burrow at an angle of about 35° to 42°, and the protruding length is about 2.6 cm.

##### Discussion

The burrow surface has distinct regular oblique ridges that can be distinguished from other species in the genus. The Shibantan materials are substantially larger than typical specimens, and adjacent burrow segments are stacked at the joint point.

##### Occurrence

The Shibantan Member of the Dengying Formation at Wuhe, Yangtze Gorges area, South China.

## MATERIALS AND METHODS

### Specimen collection and repository information

Most fossil materials derive from three horizons in the basal (0 to 5 m), lower (18 to 25 m), and middle (about 70 m) Shibantan Member around Wuhe village (30° 47′ 10″ to 30° 47′ 27″ N, 111° 02′ 47″ to 111° 03′ 23″ E) in the Yangtze Gorges area, southern China. All specimens carry unambiguous stratigraphic provenance. All cataloged specimens are reposited at the Nanjing Institute of Geology and Palaeontology, Chinese Academy of Sciences.

### Photography and data acquisition

We observed specimens both in situ and under laboratory conditions. Field and indoor photography used a Nikon D850 camera with natural and controlled lighting. Morphometric data were acquired using ImageJ 1.52v, and fossil illustrations were prepared in Photoshop CS.

## Supplementary Materials

### This PDF file includes:

Provenance Systematic paleontology Fig. S1 References
