# Supplementary material for: Advent of three-dimensional sediment exploration reveals Ediacaran-Cambrian ecosystem transition
Source: Sci Adv. 2025 Oct 29;11(44):eadx9449. doi: 10.1126/sciadv.adx9449 (PMC12571085; doi:10.1126/sciadv.adx9449)
Supplement: Supplementary file 1 — Provenance Systematic paleontology Fig. S1 References [file sciadv.adx9449_sm.pdf]

Science Advances
AAAS

Supplementary Materials for

Advent of three-dimensional sediment exploration reveals Ediacaran-Cambrian ecosystem transition

Zhe Chen and Yarong Liu

Corresponding author: Zhe Chen, zhechen@nigpas.ac.cn

Sci. Adv. **11**, eadx9449 (2025)
DOI: 10.1126/sciadv.adx9449

This PDF file includes:

Provenance
Systematic paleontology
Fig. S1
References

Provenance

Collection Details

- **Location**: Wuhe Village, Yangtze Gorges, South China (30°47′10″–30°47′27″N, 111°02′47″–111°03′23″E)
- **Horizons**: Shibantan Member, Dengying Formation:
  - Basal (0–5 m)
  - Lower (18–25 m)
  - Middle (~70 m)
- **Collectors**: Z. Chen, Y, Liu, C. Wu and X. Wang, during 2018–2024.
- **Material**: Ediacara-type body fossils and trace fossils (*Treptichnus*, *Lamonte*, tadpole-like traces)

Validation & Dating

- **Authentication**:
  - Morphological validation via comparative analysis with published taxa (*18-23*)
  - Thin-section petrography to confirm taphonomic context.
- **Dating**:
  - **U-Pb zircon geochronology** on interbedded ash layers:
    - 550.1 ± 0.6 Ma (Miaohe Member ash, equivalent to lower Shibantan) (*28*)
    - 543.4 ± 3.5 Ma (middle Baimatuo Member) (*29*)
  - **Biostratigraphy**: Ediacara-type fossils (*18, 27*), *Cloudina*/*Sinotubulites* ranges: >547.36–538.58 Ma (*16, 17, 33, 34*).

Repository & Access

- **Institution**: Nanjing Institute of Geology and Palaeontology, Chinese Academy of Sciences (NIGPAS).
- **Accession Numbers**: NIGP-205651–205655
- **Access Policy**:
  - Specimens freely accessible for research by appointment
  - No restrictions for academic/non-commercial use

Systematic paleontology

Treptichnus

(Fig. 2)

Type ichnospecies: *T. bifurcus*

Diagnosis: Simple or zigzag, straight or curved segments associated with vertical or oblique tubes comprising a three-dimensional burrow system. Joined points of segments exhibit small pits or short twig-like projections. [from (*9*)].

Discussion: The Shibantan materials are substantially larger than typical specimens, and adjacent burrow segments overlap at the joint point. Three species can be identified, including *T.* cf. *bifurcus*, *T. rectangular*, *T.* isp., and *T. streptosus* isp. nov.

Ethology: *Treptichnus* reflects successive probing activities (*44, 45*), and represents a combination of dwelling and feeding behaviors (*80*).

Occurrence: *Treptichnus* shows broad environmental tolerance and long stratigraphic distribution (*10*). The earliest report of treptichnids comes from the late Ediacaran Dunfee Member of the Deep Spring Formation in Nevada (*15*). The first appearance of *T. pedum* marks the Ediacaran-Cambrian boundary (*11*). Whereas, *T. pedum* was found several meters below the basal Cambrian GSSP in Newfoundland (*12*).

Tracemaker: *Treptichnus* can be produced by priapulid-like scalidophoran worms (*44, 45*), insect larvae (*46-48*), and insects (*49*).

***Treptichnus streptosus* isp. nov.**

(Fig. 2A)

Diagnosis: Subhorizontal burrow composed of successively superimposed burrow segments, with short projections. The burrow surface shows distinctive regular inclined ridges.

Holytype: NIGP-205651, Fig. 2A

Etymology: From the Greek *streptos*, meaning twisted.

Description: The specimen morphology shows a certain degree of regularity. The subhorizontal burrow is composed of four successively superimposed burrow segments. The burrow is slightly curved, about 10 mm in width, and 36 cm in length. The burrow

segments are slightly curved, with nearly constant length, about 11 cm. The projections protrude from the concave side of the burrow at an angle of about 35–42°, and the protruding length is about 2.6 cm.

Discussion: The burrow surface has distinct regular oblique ridges that can be distinguished from other species in the genus. The Shibantan materials are substantially larger than typical specimens in this genus, and adjacent burrow segments overlap at the joint point.

Occurrence: The Shibantan Member of the Dengying Formation at Wuhe, Yangtze Gorges area, South China.

Treptichnus* cf. *bifurcus

(Fig. 2, C to E)

Description: straight or meandering subhorizontal zigzagging burrow consists of serial burrow segments. The tube is smooth with short projections at the joint of elongated burrow segments. The burrow segment is nearly constant in width, slightly expanding at both ends. The angles between the projection and the tube body are acute.

One specimen (Fig. 2E) is meandering, about 19 cm in preserved length. It is composed of at least 10 successively burrow segments. Individual burrow segments are nearly equal in width, about 1.5–2 mm. The segments are variable in length. Burrow segments are straight or meandering with slightly expanding ends. The projection angles vary, ranging from 30° to 70°.

The other specimen (Fig. 2, C and D) is a straight subhorizontal zigzagging burrow. It is relatively large, about 65 cm in preserved length and nearly 9 mm in width. The tube is relatively smooth, with occasional inclined ridges. It is composed of six successively superimposed burrow segments. Individual burrow segments are nearly equal in length and width, about 6–9 mm in width, 12–15 cm in length. Burrow segments are slightly curved with slightly expanding ends. The angles between successive segments vary, ranging from 20° to 66°, usually at a smaller angle.

Discussion: *T. bifurcus* is diagnostically characterized by short twig-like projections at the angle of juncture between elongated, horizontal and thin burrow segments;

projections situated on alternate sides of trace (*9*). While the Shibantan specimens exhibit comparable projections and horizontal burrow segments, their probe alternation displays significantly greater irregularity and spatial chaos compared to the tightly organized, rhythmic pattern definitive of *T. bifurcus* (*9, 41*). Consequently, we assign these specimens to *Treptichnus* cf. *bifurcus* to denote morphological affinity while acknowledging key deviations.

Treptichnus pollardi

(Fig. 2H, fig. S1)

Diagnosis: *Treptichnus* having small pits either at the angle of juncture between horizontal burrow segments or within burrow segments; pits are the bedding plane expression of vertical shafts of the burrow system[from (*9*)].

Description: The trace fossil is preserved as full relief. The gross morphology of the trace is curved, showing horizontal cylindrical burrow fragments and bulbs connected alternately. The trace can be considered as a series of segments joined (as illustrated in Fig. S1C). Each segment consists of horizontal cylindrical burrow and expanded bulbous structure termination. The horizontal burrows are typically narrower than the bulbous structure in width. Each burrow is straight or curved. All the burrows are consistently about 2.5 mm in width. The bulbous structures are round or ovate, about 3.5–5.0 mm (average 4.2 mm) in width, and deeper than the remainder of the burrow. The length of segments is variable, between about 20 to 80 mm.

The preserved trace is composed of eight segments. As illustrated showing (Fig.S1C), segments (1st –7th) are connected end to end. The adjacent segments change their extending directions at the segment junctures, showing a zigzag pattern without distinctive side projection. The angle between successive segments is relatively high, about 130–160°. It's noted that segment 8th (yellow segment in Fig.S1C) seems to be a branch segment, offsetting from the burrow fragment of segment 6th.

Treptichnus rectangularis

(Fig. 2F)

Diagnosis: Horizontal burrows consisting of a system of short units, 3–5 mm in diameter and up to 40 mm long each, emerging alternately or on the same side from the periapertural part of the preceding unit and opening vertically to the surface. Units are irregularly shaped and almost devoid of ornamentation, they were created parallel and right below the water/sediment interface. The walls of burrows were stiffened; the organisms produced fecal pellets 0.3 mm in diameter and up to 1 mm long. [from (*42*)]

Description: Horizontal zigzag burrows consisting of successively connected burrow segments. The burrows are slender and slightly curved, nearly constant width, about 1.5–2.5 mm and 27 cm long. The angle of connected burrow segments is variable and tends to be right-angled. The ends of the burrow segments are expanding. The short burrow segments are commonly smooth, and long segments show declined ridges.

***Treptichnus* isp.**

(Fig. 2G)

Description: A burrow structure formed by an imbricated arrangement of four short, fusiform (spindle-shaped) segments, with no obvious protrusions. The trace fossil is preserved at 42 mm in total length and approximately 3 mm in width. Each segment measures 10–14 mm in length, with adjacent segments overlapping by roughly 3 mm.

Lamonte

Diagnosis: A network of millimeter-sized surface tracks and trails (parallel series of sharp scratch marks or shallow pits), horizontal lined burrows (composed of straight to gently curved elements filled with intraclasts and cements), and vertical traces found in association with crinkled and microlaminated layers. [from (*21*)]

Lamonte trevallis

(Fig.3, A and B)

Description: The trace fossil consists of three different morphotypes (horizontal

burrows, surface trails, and vertical traces) that are sometimes connected. Surface trails are relatively rare, while bilobate horizontal burrows frequently connect with vertical burrows. The burrows may branch from either horizontal or vertical burrows, often overlapping one another but rarely cross-cutting. Vertical burrows are typically located at the ends of horizontal burrows, though they occasionally appear in the middle of horizontal traces. The horizontal burrows exhibit a bilobed transverse cross-section, with a straight to gently curved morphology and a width ranging from 3 to 8 mm. In contrast, the vertical burrows have a larger diameter than the horizontal burrows and feature a spherical base with a slight central dimple.

tadpole-like trace

(Fig. 3, C to F)

Description: The trace consists of a narrow burrow and expanding bulb structure, resembling the shape of a tadpole. The fossils can be divided into two parts: a spherical bulbous end and a slender burrow. The bulbous end is nearly spherical, with a diameter ranging from approximately 5 to 12 mm. The burrow is horizontal or subhorizontal, and is either straight or curved. The burrow is connected to the bulbous end and tapers toward its distal end, with a maximum diameter of about 3–7 mm. The surface of the fossils is smooth, with no visible ornamentation.

The fossils are preserved in full relief, with the majority appearing as positive convex features on the bottom surface of the limestone layer and corresponding concave impressions on the top surface. The fossils are scattered across the bedding plane without a clear orientation and rarely overlap or cross-cut each other.

In the thin section (Fig. 2F), the fossils and the surrounding rock are primarily composed of carbonate. The burrow appears brighter than the surrounding matrix due to a higher concentration of sparry cement. The fossils are located between microbial mat layers and are oriented parallel to these layers. The boundaries between the fossils, the surrounding rock, and the microbial mats are indistinct, with no visible lining structures or active filling structures, such as meniscus structures. The bulbous end truncates the original microbial mat layers, and the microbial mat layers in the bulbous

region are noticeably thinner than in other areas.

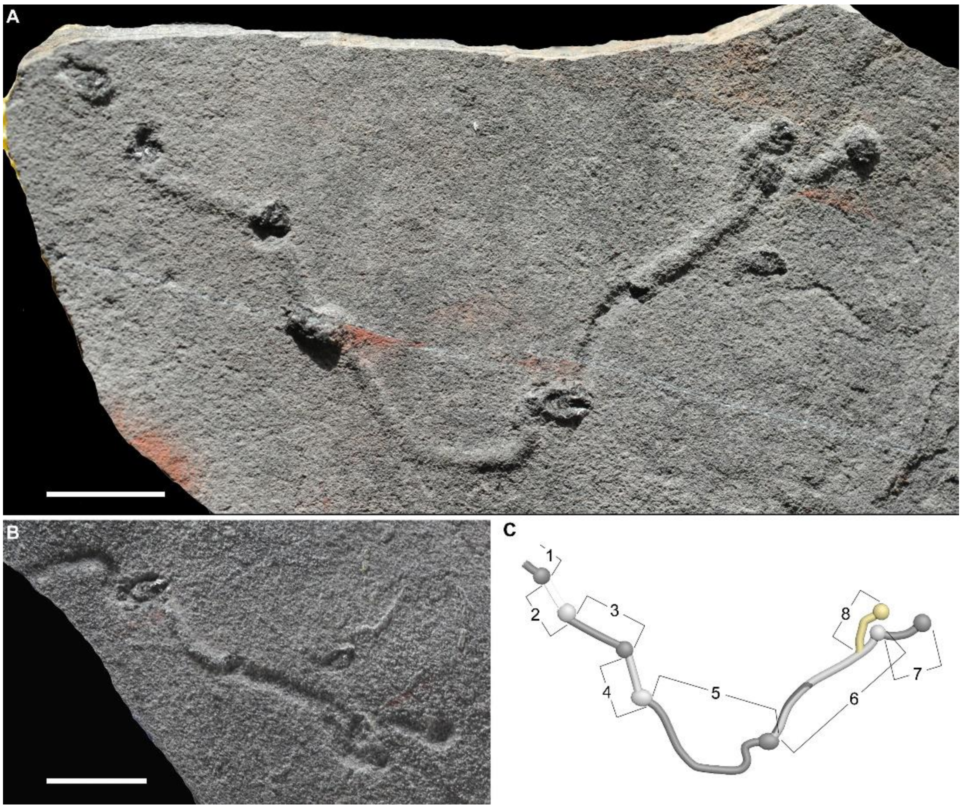

Supplementary Fig. 1. *Treptichnus pollardi* trace found in the Dengying Formation in Wuhe. (A) to (B) part and counterpart, showing burrow segments and termination bulbous structure arranged in zigzag shape, NIGP-205655; (C) Illustration of Treptichnus, noting possible branch (segment 8th in yellow), Illustration by Z. Chen. Scale bars represent 10 mm.

REFERENCES AND NOTES

1. A. T. Cribb, C. G. Kenchington, B. Koester, B. M. Gibson, T. H. Boag, R. A. Racicot, H. Mocke, M. Laflamme, S. A. F. Darroch, Increase in metazoan ecosystem engineering prior to the Ediacaran-Cambrian boundary in the Nama Group, Namibia. *R. Soc. open sci.* **6**, 190548 (2019).

2. M. G. Mángano, L. A. Buatois, The rise and early evolution of animals: Where do we stand from a trace-fossil perspective? *Interface Focus* **10**, 20190103 (2020).

3. S. Jensen, The Proterozoic and earliest Cambrian trace fossil record: Patterns, problems and perspectives. *Integr. Comp. Biol.* **43**, 219–228 (2003).

4. M. G. Mángano, L. A. Buatois, Decoupling of body-plan diversification and ecological structuring during the Ediacaran–Cambrian transition: Evolutionary and geobiological feedbacks. *Proc. R. Soc. B* **281**, 20140038 (2014).

5. M. G. Mángano, L. A. Buatois, The Cambrian revolutions: Trace-fossil record, timing, links and geobiological impact. *Earth Sci. Rev.* **173**, 96–108 (2017).

6. S. Jensen, T. Palacios, The Ediacaran-Cambrian trace fossil record in the Central Iberian Zone, Iberian Peninsula. *Comun. Geol.* **103**, 83–92 (2016).

7. D. McIlroy, M. D. Brasier, "Ichnological evidence for the Cambrian explosion in the Ediacaran to Cambrian succession of Tanafjord, Finnmark, northern Norway," in *Earth System Evolution and Early Life: A Celebration of the Work of Martin Brasier,* A. T. Brasier, D. McIlroy, N. McLoughlin, Eds. (Geological Society of London, 2016), vol. 448, pp. 351–368.

8. L. A. Buatois, J. Almond, G. J. B. Germs, Environmental tolerance and range offset of *Treptichnus pedum*: Implications for the recognition of the Ediacaran-Cambrian boundary. *Geology* **41**, 519–522 (2013).

9. L. A. Buatois, M. G. Mángano, The ichnotaxonomic status of *Plangtichnus* and *Treptichnus*. *Ichnos* **2**, 217–224 (1993).

10. L. A. Buatois, *Treptichnus pedum* and the Ediacaran–Cambrian boundary: Significance and caveats. *Geol. Mag.* **155**, 174–180 (2017).

11. G. M. Narbonne, P. M. Myrow, E. Landing, M. M. Anderson, A candidate stratotype for the Precambrian-Cambrian boundary, Fortune Head, Burin Peninsula, southeastern Newfoundland. *Can. J. Earth Sci.* **24**, 1277–1293 (1987).

12. J. G. Gehling, S. Jensen, M. L. Droser, P. M. Myrow, G. M. Narbonne, Burrowing below the basal Cambrian GSSP, Fortune Head, Newfoundland. *Geol. Mag.* **138**, 213–218 (2001).

13. S. Jensen, B. Z. Saylor, J. G. Gehling, G. J. B. Germs, Complex trace fossils from the terminal Proterozoic of Namibia. *Geology* **28**, 143–146 (2000).

14. S. A. F. Darroch, A. T. Cribb, L. A. Buatois, G. J. B. Germs, C. G. Kenchington, E. F. Smith, H. Mocke, G. R. O'Neil, J. D. Schiffbauer, K. M. Maloney, R. A. Racicot, K. A. Turk, B. M. Gibson, J. Almond, B. Koester, T. H. Boag, S. M. Tweedt, M. Laflamme, The trace fossil record of the Nama Group, Namibia: Exploring the terminal Ediacaran roots of the Cambrian explosion. *Earth Sci. Rev.* **212**, 103435 (2021).

15. L. G. Tarhan, P. M. Myrow, E. F. Smith, L. L. Nelson, P. M. Sadler, Infaunal augurs of the Cambrian explosion: An Ediacaran trace fossil assemblage from Nevada, USA. *Geobiology* **18**, 486–496 (2020).

16. L. L. Nelson, J. Ramezani, J. E. Almond, S. A. F. Darroch, W. L. Taylor, D. C. Brenner, R. P. Furey, M. Turner, E. F. Smith, Pushing the boundary: A calibrated Ediacaran-Cambrian stratigraphic record from the Nama Group in northwestern Republic of South Africa. *Earth Planet. Sci. Lett.* **580**, 117396 (2022).

17. Z. Gong, I. Baillie, L. L. Nelson, S. H. Gerasimov, C. Rose, E. F. Smith, Magnetic susceptibility cyclostratigraphy of the lower Schwarzrand Subgroup in southern Namibia refines temporal calibration of late Ediacaran bilaterian radiation. *Global Planet. Change* **245**, 104668 (2025).

18. S. H. Xiao, Z. Chen, K. Pang, C. M. Zhou, X. L. Yuan, The Shibantan Lagerstatte: Insights into the Proterozoic-Phanerozoic transition. *J. Geol. Soc.* **178**, jgs2020-135 (2021).

19. Z. Chen, C. Zhou, M. Meyer, K. Xiang, J. D. Schiffbauer, X. Yuan, S. Xiao, Trace fossil evidence for Ediacaran bilaterian animals with complex behaviors. *Precambrian Res.* **224**, 690–701 (2013).

20. X. Chen, X. Yuan, C. Zhou, Z. Chen, Ediacaran tadpole-like trace fossils from the Dengying Formation in the Yangtze Gorges area, Hubei Province. *Acta Palaeontol. Sin.* **57**, 1–10 (2018).

21. M. Meyer, S. Xiao, B. C. Gill, J. D. Schiffbauer, Z. Chen, C. Zhou, X. Yuan, Interactions between Ediacaran animals and microbial mats: Insights from *Lamonte trevallis*, a new trace fossil from the Dengying Formation of South China. *Palaeogeogr. Palaeoclimatol. Palaeoecol.* **396**, 62–74 (2014).

22. Z. Chen, C. Zhou, S. Xiao, W. Wang, C. Guan, H. Hua, X. Yuan, New Ediacara fossils preserved in marine limestone and their ecological implications. *Sci. Rep.* **4**, 4180 (2014).

23. X. P. Wang, Z. Chen, K. Pang, C. Zhou, S. Xiao, B. Wan, X. Yuan, *Dickinsonia* from the Ediacaran Dengying Formation in the Yangtze Gorges area, South China. *Palaeoworld* **30**, 602–609 (2021).

24. G. Jiang, X. Shi, S. Zhang, Y. Wang, S. Xiao, Stratigraphy and paleogeography of the Ediacaran Doushantuo Formation (ca. 635–551 Ma) in South China. *Gondwana Res.* **19**, 831–849 (2011).

25. L. Dong, S. Xiao, B. Shen, C. Zhou, G. Li, J. Yao, Basal Cambrian microfossils from the Yangtze Gorges area (South China) and the Aksu area (Tarim Block, northwestern China). *J. Paleontol.* **83**, 30–44 (2009).

26. M. Steiner, G. Li, Y. Qian, M. Zhu, B. D. Erdtmann, Neoproterozoic to Early Cambrian small shelly fossil assemblages and a revised biostratigraphic correlation of the Yangtze Platform (China). *Palaeogeogr. Palaeoclimatol. Palaeoecol.* **254**, 67–99 (2007).

27. A. D. Muscente, N. Bykova, T. H. Boag, L. A. Buatois, M. G. Mangano, A. Eleish, A. Prabhu, F. Pan, M. B. Meyer, J. D. Schiffbauer, P. Fox, R. M. Hazen, A. H. Knoll, Ediacaran biozones identified with network analysis provide evidence for pulsed extinctions of early complex life. *Nat. Commun.* **10**, 911 (2019).

28. C. Yang, A. D. Rooney, D. J. Condon, X.-H. Li, D. V. Grazhdankin, F. T. Bowyer, C. Hu, F. A. Macdonald, M. Zhu, The tempo of Ediacaran evolution. *Sci. Adv.* **7**, eabi9643 (2021).

29. T. Huang, D. Chen, Y. Ding, X. Zhou, G. Zhang, SIMS U-Pb zircon geochronological and carbon isotope chemostratigraphic constraints on the Ediacaran-Cambrian boundary succession in the Three Gorges area, South China. *J. Earth Sci.* **31**, 69–78 (2020).

30. Z. Zhao, Y. Xing, Q. Ding, G. Liu, Y. Zhao, S. Zhang, X. Meng, C. Yin, B. Ning, P. Han, Eds., *The Sinian System of Hubei* (China University of Geosciences Press, Wuhan, 1988).

31. Z. An, G. Jiang, J. Tong, L. Tian, Q. Ye, H. Song, H. Song, Stratigraphic position of the Ediacaran Miaohe biota and its constrains on the age of the upper Doushantuo $\delta^{13}C$ anomaly in the Yangtze Gorges area, South China. *Precambrian Res.* **271**, 243–253 (2015).

32. C. Zhou, Q. Ouyang, W. Wang, B. Wan, C. Guan, Z. Chen, X. Yuan, Lithostratigraphic subdivision and correlation of the Ediacaran in China. *J. Stratigr.* **45**, 211–222 (2021).

33. J. P. Grotzinger, S. A. Bowring, B. Z. Saylor, A. J. Kaufman, Biostratigraphic and geochronologic constraints on early animal evolution. *Science* **270**, 598–604 (1995).

34. U. Linnemann, M. Ovtcharova, U. Schaltegger, A. Gärtner, M. Hautmann, G. Geyer, P. Vickers-Rich, T. Rich, B. Plessen, M. Hofmann, J. Zieger, R. Krause, L. Kriesfeld, J. Smith, New high-resolution age data from the Ediacaran–Cambrian boundary indicate rapid, ecologically driven onset of the Cambrian explosion. *Terra Nova* **31**, 49–58 (2019).

35. L. Zhang, S. Chang, C. Chen, X. Wang, Q. Feng, M. Steiner, B. Yang, R. Mason, Z. She, J. Yan, J. Vannier, M.-B. Forel, Q. Xiao, S. Clausen, *Cloudina* aggregates from the uppermost Dengying Formation, Three Gorges area, South China, and stratigraphical implications. *Precambrian Res.* **370**, 106552 (2022).

36. D. Liang, Y. Cai, M. Nolan, S. Xiao, The terminal Ediacaran tubular fossil *Cloudina* in the Yangtze Gorges area of South China. *Precambrian Res.* **351**, 105931 (2020).

37. M. Chen, Y. Chen, Y. Qian, Some tubular fossils from Sinian-Lower Cambrian boundary sequences, Yangtze Gorge. *Bull. of Tianjin Inst. Geol. M. R., Chinese Acad. Geol. Sci.* **3**, 117–124 (1981).

38. X. H. Chen, P. Zhou, B. M. Zhang, K. Wei, M. Zhang, Lithostratigraphy, biostratigraphy, sequencestratigraphy and carbon isotope chemostratigraphy of the upper Ediacarian in Yangtze Gorges and their significance for choronostratigraphy. *Geol. Miner. Resour. South China* **32**, 87–105 (2016).

39. X. Wang, B. Yang, Z. An, Z. Zhao, Taphonomic analysis of the *Sinotubulites* from the Shibantan Member of the Dengying Formation in Yangtze Gorges Area (China). *Minerals* **15**, 570 (2025).

40. P. R. Getty, T. D. McCarthy, S. Hsieh, A. M. Bush, A new reconstruction of continental *Treptichnus* based on exceptionally preserved material from the Jurassic of Massachusetts. *J. Paleontol.* **90**, 269–278 (2016).

41. S. R. Hammersburg, S. T. Hasiotis, R. A. Robison, Ichnotaxonomy of the Cambrian Spence Shale Member of the Langston Formation, Wellsville Mountains, Northern Utah, USA. *Paleontol. Contrib.* **20**, 1–66 (2018).

42. S. Orłowski, A. Żylińska, Non-arthropod burrows from the Middle and Late Cambrian of the Holy Cross Mountains, Poland. *Acta Palaeontol. Pol.* **41**, 385–409 (1996).

43. S. Jensen, B. N. Runnegar, A complex trace fossil from the Spitskop Member (terminal Ediacaran -? Lower Cambrian) of southern Namibia. *Geol. Mag.* **142**, 561–569 (2005).

44. J. Vannier, I. Calandra, C. Gaillard, A. Zylinska, Priapulid worms: Pioneer horizontal burrowers at the Precambrian-Cambrian boundary. *Geology* **38**, 711–714 (2010).

45. K. A. Turk, A. Wehrmann, M. Laflamme, S. A. F. Darroch, Priapulid neoichnology, ecosystem engineering, and the Ediacaran–Cambrian transition. *Palaeontology* **67**, e12721 (2024).

46. A. K. Rindsberg, D. C. Kopaska-Merkel, "*Treptichnus* and *Arenicolites* from the Steven C. Minkin Paleozoic footpreint site (Langsettlan, Alabama, USA)," in *Pennsylvanian Footprints in the Black Warrior Basin of Alabama. Alabama Paleontological Society Monograph no. 1.,* R. J. Buta, A. K. Rindsberg, D. C. Kopaska-Merkel, Eds. (Alabama Paleontological Society, Birmingham, 2005), pp. 121–141.

47. J. H. D. Lima, N. J. Minter, R. G. Netto, Insights from functional morphology and neoichnology for determining tracemakers: A case study of the reconstruction of an ancient glacial arthropod-dominated fauna. *Lethaia* **50**, 576–590 (2017).

48. A. Uchman, "*Treptichnus*-like traces made by insect larvae (Diptera: Chironomidae, Tipulidae)" in *Pennsylvanian Footprints in the Black Warrior Basin of Alabama. Alabama Paleontological Society Monograph no. 1.,* R. J. Buta, A. K. Rindsberg, D. C. Kopaska-Merkel, Eds. (Alabama Paleontological Society, Birmingham, 2005), pp. 143–146.

49. A. Baucon, A. Ronchi, F. Felletti, C. N. de Carvalho, Evolution of Crustaceans at the edge of the end-Permian crisis: Ichnonetwork analysis of the fluvial succession of Nurra (Permian-Triassic, Sardinia, Italy). *Palaeogeogr. Palaeoclimatol. Palaeoecol.* **410**, 74–103 (2014).

50. C. Carbone, G. M. Narbonne, When life got smart: The evolution of behavioral complexity through the Ediacaran and Early Cambrian of NW Canada. *J. Paleontol.* **88**, 309–330 (2014).

51. M. Bertling, S. J. Braddy, R. G. Bromley, G. R. Demathieu, J. Genise, R. Mikuláš, J. K. Nielsen, K. S. Nielsen, A. K. Rindsberg, M. Schlirf, Names for trace fossils: A uniform approach. *Lethaia* **39**, 265–286 (2006).

52. L. R. Menon, D. McIlroy, M. D. Brasier, Evidence for Cnidaria-like behavior in ca. 560 Ma Ediacaran. *Geology* **41**, 895–898 (2013).

53. A. G. Liu, D. Mcilroy, J. J. Matthews, M. D. Brasier, Confirming the metazoan character of a 565 Ma trace-fossil assemblage from Mistaken Point, Newfoundland. *Palaios* **29**, 420–430 (2014).

54. A. G. Liu, D. McIlroy, M. D. Brasier, First evidence for locomotion in the Ediacara biota from the 565 Ma Mistaken Point Formation, Newfoundland. *Geology* **38**, 123–126 (2010).

55. A. Seilacher, Ed., *Trace Fossil Analysis*. (Springer Berlin Heidelberg, Berlin, 2007).

56. L. A. Buatois, M. G. Mángano, "Ediacaran ecosystems and the dawn of animals," in *The Trace-Fossil Record of Major Evolutionary Events: Volume 1: Precambrian and Paleozoic,* M. G. Mángano, L. A. Buatois, Eds. (Springer Netherlands, Dordrecht, 2016), pp. 27–72.

57. S. Jensen, M. L. Droser, J. G. Gehling, "A critical look at the Ediacaran trace fossil record," in *Neoproterozoic Geobiology*, S. Xiao, A. J. Kaufman, Eds. (Kluwer Academic Publishers, Dordrecht, Netherlands, 2006), pp. 115–157.

58. A. Seilacher, L. A. Buatois, M. Gabriela Mangano, Trace fossils in the Ediacaran-Cambrian transition: Behavioral diversification, ecological turnover and environmental shift. *Palaeogeogr. Palaeoclimatol. Palaeoecol.* **227**, 323–356 (2005).

59. B. A. Laing, M. G. Mángano, L. A. Buatois, G. M. Narbonne, R. C. Gougeon, A protracted Ediacaran–Cambrian transition: An ichnologic ecospace analysis of the Fortunian in Newfoundland, Canada. *Geol. Mag.* **156**, 1623–1630 (2019).

60. B. A. Laing, L. A. Buatois, M. G. Mángano, N. J. Minter, L. C. Strotz, G. M. Narbonne, G. A. Brock, Bioturbators as ecosystem engineers: Assessing current models. *Palaios* **37**, 718–730 (2022).

61. E. G. Mitchell, S. Pates, From organisms to biodiversity: The ecology of the Ediacaran/Cambrian transition. *Paleobiology* **51**, 150–173 (2025).

62. G. Mussini, F. S. Dunn, Decline and fall of the Ediacarans: Late-Neoproterozoic extinctions and the rise of the modern biosphere. *Biol. Rev.* **99**, 110–130 (2024).

63. L. G. Herringshaw, R. H. T. Callow, D. McIlroy, "Engineering the Cambrian explosion: The earliest bioturbators as ecosystem engineers," in *Earth System Evolution and Early Life: A Celebration of the Work of Martin Brasier*, A. T. Brasier, D. McIlroy, N. McLoughlin, Eds. (Geological Society of London, 2017), vol. 448, pp. 369–382.

64. A. T. Cribb, S. J. van de Velde, W. M. Berelson, D. J. Bottjer, F. A. Corsetti, Ediacaran–Cambrian bioturbation did not extensively oxygenate sediments in shallow marine ecosystems. *Geobiology* **21**, 435–453 (2023).

65. D. E. Canfield, J. Farquhar, Animal evolution, bioturbation, and the sulfate concentration of the oceans. *Proc. Natl. Acad. Sci. U.S.A.* **106**, 8123–8127 (2009).

66. R. A. Boyle, T. W. Dahl, C. J. Bjerrum, D. E. Canfield, Bioturbation and directionality in Earth's carbon isotope record across the Neoproterozoic–Cambrian transition. *Geobiology* **16**, 252–278 (2018).

67. L. G. Tarhan, M. Zhao, N. J. Planavsky, Bioturbation feedbacks on the phosphorus cycle. *Earth Planet. Sci. Lett.* **566**, 116961 (2021).

68. D. H. Erwin, S. Tweedt, Ecological drivers of the Ediacaran-Cambrian diversification of Metazoa. *Evol. Ecol.* **26**, 417–433 (2012).

69. A. Seilacher, F. Pflüger, "From biomats to benthic agriculture: A biohistoric revolution" in *Biostabilization of Sediments,* W. S. Krumbein, D. M. Paterson, L. S. Stal, Eds. (Biblioteks und Informationsystem der Universität Oldenburg, Oldenburg, Germany, 1994), pp. 97–105.

70. S. D. Evans, C. Tu, A. Rizzo, R. L. Surprenant, P. C. Boan, H. McCandless, N. Marshall, S. Xiao, M. L. Droser, Environmental drivers of the first major animal extinction across the Ediacaran White Sea-Nama transition. *Proc. Natl. Acad. Sci. U.S.A.* **119**, e2207475119 (2022).

71. S. A. F. Darroch, E. F. Smith, L. L. Nelson, M. Craffey, J. D. Schiffbauer, M. Laflamme, Causes and consequences of end-Ediacaran extinction: An update. *Cambridge Prisms Extinct.* **1**, e15 (2023).

72. S. A. F. Darroch, E. F. Smith, M. Laflamme, D. H. Erwin, Ediacaran extinction and Cambrian explosion. *Trends Ecol. Evol.* **33**, 653–663 (2018).

73. M. Craffey, P. J. Wagner, D. K. Watkins, S. A. F. Darroch, S. K. Lyons, Co-occurrence structure of late Ediacaran communities and influence of emerging ecosystem engineers. *Proc. R. Soc. B* **291**, 20242029 (2024).

74. E. B. Hodgin, L. L. Nelson, C. J. Wall, A. J. Barrón-Díaz, L. C. Webb, M. D. Schmitz, D. A. Fike, J. W. Hagadorn, E. F. Smith, A link between rift-related volcanism and end-Ediacaran extinction? Integrated chemostratigraphy, biostratigraphy, and U-Pb geochronology from Sonora, Mexico. *Geology* **49**, 115–119 (2020).

75. D. H. Erwin, Macroevolution of ecosystem engineering, niche construction and diversity. *Trends Ecol. Evol.* **23**, 304–310 (2008).

76. E. G. Mitchell, N. Bobkov, N. Bykova, A. Dhungana, A. V. Kolesnikov, I. R. P. Hogarth, A. G. Liu, T. M. R. Mustill, N. Sozonov, V. I. Rogov, S. Xiao, D. V. Grazhdankin, The influence of environmental setting on the community ecology of Ediacaran organisms. *Interface Focus* **10**, 20190109 (2020).

77. K. A. Turk, M. A. Pulsipher, H. Mocke, M. Laflamme, S. A. F. Darroch, *Himatiichnus mangano* igen. et isp. nov., a scalidophoran trace fossil from the late Ediacaran of Namibia. *R. Soc. open sci.* **11**, 240452 (2024).

78. J. G. Gehling, M. L. Droser, Ediacaran scavenging as a prelude to predation. *Emerg. Top. Life Sci.* **2**, 213–222 (2018).

79. M. D. Schmitz, “Appendix 2—Radiometric ages used in GTS2012,” in *The Geologic Time Scale 2012,* F. Gradstein, J. Ogg, M. D. Schmitz, G. Ogg, Eds. (Elsevier, Boston, 2012), pp. 1045–1082.

80. L. A. Buatois, M. G. Mangano, Eds., *Ichnology: Organism-Substrate Interactions in Space And Time.* (Cambridge Univ. Press, Cambridge, 2011).
